# A cross-sectional exploratory analysis between pet ownership, sleep, exercise, health and neighbourhood perceptions: the Whitehall II cohort study

**DOI:** 10.1186/s12877-018-0867-3

**Published:** 2018-08-09

**Authors:** Gill Mein, Robert Grant

**Affiliations:** Faculty of Health, Social Care and Education, Kingston University and St George’s, University of London, Cranmer Terrace, London, SW17 0RE UK

**Keywords:** Sleep, Pets, Dogs, Exercise, Neighbourhood perceptions

## Abstract

**Background:**

To explore associations between pets, and specifically dog ownership and sleep, health, exercise and neighbourhood.

**Methods:**

Cross sectional examination of 6575 participants of the Whitehall II study aged between 59 and 79 years. We used self-assessed measurement scales of the Short Form (SF36), General Health Questionnaire (GHQ), Control, Autonomy, Self-realisation and Pleasure (CASP), Centre for Epidemiologic Studies Depression Scale (CES-D), sleep, exercise, and perceptions of local neighbourhood. In addition the Mini Mental State Examination which is administered to test global cognitive status (MMSE).

**Results:**

We found 2/7 people owned a pet and of those 64% were “very” attached to their pet. Mild exercise in metabolic equivalents (MET-hours) was significantly higher in pet owners than non-owners (median 27.8 (IQR 18.1 to 41.8) vs 25.7 (IQR 16.8 to 38.7), *p* = 0.0001), and in dog owners than other pets (median 32.3 (IQR 20.8 to 46.1) vs 25.6 (IQR 16.8 to 38.5), *p* < 0.0001). Moderate exercise was also significantly higher in pet owners than non pet owners (median 11.8 (IQR 4.2 to 21.9) vs 9.8 (IQR 2.8 to 19.5), *p* < 0.0001), and dog owners than owners of other pets (median 12.3 (IQR 4.2 to 22.2) vs 10.1 (3.1 to 20.0), *p* = 0.0002) but there were no significant differences with vigorous exercise. We found that pet owners were significantly more positive about their neighbourhood than non-owners on 8/9 questions, while dog owners were (significantly) even more positive than owners of other pets on 8/9 questions. Associations with sleep were mixed, although dog owners had less trouble falling asleep than non-dog owners, with borderline statistical significance.

**Conclusion:**

Dog owners feel more positive about their neighbourhood, do more exercise, and fall asleep more easily than non-dog owners. These results suggest that dog owners could be more likely to exercise by walking their dogs and therefore may be more familiar and positive about the area in which they walk their dog.

## Background

Researchers frequently claim there are health and social benefits from interacting with pets, particularly dogs [1]. The suggested health benefits include intrinsic benefits of lowering blood pressure [2, 3] reducing medication input, fewer visits to the doctor [4, 5] and lowering the risk of heart disease or dying within a year of having a heart attack [6]. Owning a pet has also been found to improve self-esteem, reduce stress [5, 6] and provide support for women suffering from loneliness [7, 8]. However, there are also contradictory results with pet owners aged 60–64 reporting poorer physical and mental health and a higher use of pain relief medication [9]. McNicholas et al. [10] commented as to whether pet ownership was advisable on health grounds in relation to the conflicting available evidence and argued that a broader definition of health that encompasses physical and mental dimensions of wellbeing and social integration is necessary.

Dog ownership increases the owners’ physical activity, and has been found to increase social interaction [11]. Knight and Edwards found from focus groups that dog owners made new friendship groups linked to owning a dog and subsequently felt their social circle had widened [12].

Toohey and Rock [13] found environment to be an important factor influencing physical activity for dog owners and non-dog owners. They particularly focused on social and physical environments and found owning a dog had the ability to increase physical activity, although their presence or absence did not affect perception of the physical and social environments of all neighbourhoods. They found that in disadvantaged neighbourhoods, the health of women as well as older adults may be adversely affected by a fear of other people’s dogs and associated anti-social behaviour. Cutt et al. [14] and Knight and Edwards [12] also found that negative environmental factors influenced physical activity, e.g. lack of public open spaces as a barrier to exercising. However, Cutt in contradiction also found walking with a pet allowed owners to feel more comfortable and safe about their neighbourhood.

However, association is not causation, and a debate persists about whether healthy people are more likely to get a pet or whether owning a pet makes them healthy [15]. In comparison, Parslow [9] suggested good physical health may be required for, and not the result of owning a pet, suggesting a reverse causality. Headley [15] also proposed subgroups of pet owners (those who actually cared for the pet) might benefit more than others. In support of Headley’s proposed economic benefits of pet ownership, Knight and Edwards [12] found dog ownership physically and psychologically beneficial for older people, in turn relieving pressure on health and social services.

The potential economic benefits of owning a pet by preventing illnesses is apparent. Headley [15] related the health benefits to the potential economic benefits of pets, using a natural experiment in urban China where a ban on owning a pet was lifted in 1992. This allowed their analysis to avoid potential confounders of previous or other pets. Since the ban was lifted there has been an increase in dog ownership particularly amongst women aged 25–40. Survey results of this group found owners recorded better health related outcomes than non-owners, including higher self-reported fitness, improved sleep, took fewer days off sick, and were seen less by doctors. This was the only article we found from a literature search that linked pet ownership and sleep.

Ageing is accompanied by a decrease in duration of good quality nocturnal sleep and changes in sleep routines [16, 17]. An analysis using the English Longitudinal Study of Ageing (ELSA, wave 4) reported poor sleep quality being strongly associated with poor self-reported health in a sample aged 50–74 years [18]. They also found that people who did not participate in exercise were more likely to report poor sleep. Reid et al. [19] reported increased activity levels in older adults improved sleep quality. These three factors can all influence each other, making causal inferences especially difficult.

Our analysis considers a new way of addressing how pets may affect the health of people, and how this in turn may affect how they view their own health. We hypothesize that, although pet ownership and health may be correlated positively or negatively, dog ownership in particular is associated with better sleep, exercise and health. In concrete terms, people who exercise their dogs do more mild and moderate activity, are more likely to sleep better, have a wider social network (especially in those lacking social activity), and have more positive perceptions of the neighbourhood. For some or all of these reasons, they are likely to report better health. Specifically, we address the following questions using the Whitehall II study phase 9 data collection (2009) of participants aged 59–79 years:

- how do older pet owners and non-owners differ in demographics and general health?
- adjusting for confounders, is there a difference in sleep and other outcomes between pet owners and non-owners?
- is any difference observed particularly evident in dog owners compared to other pets?
- to what extent does owning a pet or dog effect sleep outcomes appear to be mediated by physical exercise, or neighbourhood perceptions?
- are pet or dog effects particularly strong in people who are more socially isolated (effect modification)?

## Methods

The original sample of the Whitehall II study, which began in 1985 comprised of 10,308 civil servants working in 20 London based civil service departments. Since the study began participants have self-completed a questionnaire every two years and had a medical examination every five years. Each collection of data is called a phase and phase 9 was collected between 2007 and 2009 [20]. This analysis used cross sectional data from the self-completed questionnaire used in Phase 9 of the Whitehall II study.

The original aim of the Whitehall II study was to examine the effect of psychosocial, and work related factors on the health of participants. The ability to use participants occupational salary structure enabled examination of social class differences, and therefore inequalities in health. As participants retired from work other factors became important to them including family, hobbies and interests [21]. Following the interviews a question about pet ownership was Included in the Phase 9 questionnaire. Participants were asked whether they have a pet at home and the kind of pet they have. Responses were one or more of, a dog, cat, fish, bird, or “other”. The free-text details of the “other” pets were categorised by species and the most common were counted; the respondent’s judgement as to what constituted a pet as opposed to farm animals or wild animals was accepted as written on the questionnaire, although there were few of these. The questionnaire also asked participants how attached they felt to their pet(s).

Our primary outcome was a series of five questions on the self–completed questionnaire using the 4-item Jenkins scale (with an additional question) asking how often in the last month the participant had experienced: having trouble falling asleep, waking up several times per night, having trouble staying asleep (including waking too early), waking up after your usual amount of sleep feeling tired and worn out, and having disturbed or restless sleep [22]. We analysed each separately as an ordinal variable. We explored whether any associations with pet ownership were mediated by exercise or perceptions of the neighbourhood. Exercise was measured using questions in the self administered questionnaire and includes asking participants about their physical activity at home and at work in the previous 4 weeks using Metabolic Equivalents (METs) divided into mild, moderate or vigorous intensities [23]. Perceptions of the neighbourhood were measured by nine Likert items, each with seven values reversed when necessary so that high values indicate positive views [24, 25]:

- I really feel part of this area
- There is no problem with vandalism and graffiti in this area
- I have never felt lonely living in this area
- Most people in this area can be trusted
- People feel safe walking alone in this area after dark
- Most people in this area are friendly
- People in this area will always treat you fairly
- This area is kept very clean
- If you were in trouble, there are lots of people in this area who would help you

We measured participants health and wellbeing using separate composite scores of the physical and mental health questions in the SF36 [26]. The General Health Questionnaire is a 30 item questionnaire which measures minor psychiatric morbidity using a 4 point Likert scale [27]. Whilst depression was measured using the CES-D depression scale of 20 questions with Likert scales [28], and quality of life in old age using the CASP scale of 19 questions using a Likert scale with 4 values measuring overall wellbeing in older people independent of factors such as health [29]. Global cognitive status was tested using an administered set of questions during the medical examination. (MMSE) [30]. We measured participants’ demographics in terms of age, marital status, occupational grade, retirement and number of social activities [31].

### Statistical analysis

Associations between pet ownership and covariates or outcomes were tested using chi-squared tests for nominal variables, chi-squared tests for trend for ordinal variables, and t-tests or Wilcoxon-Mann-Whitney rank sum tests for numeric variables. The pet-sleep relationship was adjusted for potential confounding factors and mediators (as listed in Table 4) using multivariable proportional odds regression. Bootstrap confidence intervals were used in the presence of floor and ceiling effects. Bootstrap is a statistical technique for assessing uncertainty in variables that are not normally distributed [32]. In this analysis we had a number of variables with upper and lower limits to the values they can take. Standard confidence intervals can extend beyond these limits but bootstrap versions do not. Kendall’s tau-B correlations with bootstrap confidence intervals were used to examine associations between pairs of ordinal variables. As this is an exploratory study and many different comparisons and correlations were evaluated, we use *p*-values here as a metric to judge the effect size compared to the random variation in the data, but we do not draw binary (effect/no effect) conclusions from them. All analyses were conducted using Stata software, version 14.

## Results

The Phase 9 questionnaire of Whitehall II was completed by 6761 people of whom 6575 provided data on the pet questions and who form the analysis sample.

### Pet ownership

Approximately two out of every seven people (29%, 1929/6575) were pet owners: 11% (718) owned dogs, 18% (1199) cats, 4% (234) fish, 1% (68) birds and 2% (129) other. Nearly one quarter of respondents (24%) had pets in only one species category, 4% had pets in two species category, 1% in more species categories. The most common “other” pets were: rabbits (*n* = 31), tortoises (*n* = 24), horses & ponies (*n* = 16), guinea pigs (*n* = 14), chickens & poultry (*n* = 11), hamsters (*n* = 10).

When asked about their attachment, 64% were “very attached”, 24% were “fairly attached”, 9% were “slightly attached”, and 3% were “not attached” (*n* = 1929). The percentage of “very attached” owners was 78% among dog owners, 64% among cat owners, 46% among bird owners, and 41% among fish owners.

### Demographics and health

Table 1 gives demographic and basic health characteristics comparing pet owners and non-owners. We focused on dog owners and cat owners (which are not mutually exclusive) as the two most common species categories.

**Table 1:** Characteristics of participants

	Non-owners (*n* = 4646)	Pet owners (*n* = 1929)	Dog owners (*n* = 718)	Cat owners (*n* = 1199)
Demographic variables				
Mean age in years (SD)	66.4 (6.0)	64.9 (5.7)	65.1 (5.7)	64.9 (5.7)
% retired	86% (3985)	82% (1586)	83% (595)	82% (978)
% married or cohabiting	72% (3318)	82% (1569)	84% (602)	80% (961)
Occupational grade: Administrative (highest)	48% (2166/4548)	47% (880/1857)	47% (322/686)	48% (555/1153)
Occupational grade: Professional / executive (middle)	41% (1878/4548)	45% (831/1857)	48% (329/686)	43% (493/1153)
Occupational grade: Clerical / support (lowest)	11% (504/4548)	8% (146/1857)	5% (35/686)	9% (105/1153)
Mean number of social activities (median; IQR)	18.2 (18; 15 to 22)	18.9 (19; 15 to 22)	18.8 (19; 15 to 22)	18.9 (19; 15 to 23)
Health & quality of life variables				
Long-standing illness (self-reported at any time)	66%	64%	65%	64%
Mean CASP quality of life score (median; IQR)	43.2 (45; 39 to 49)	42.7 (44; 38 to 49)	42.5 (44; 38 to 49)	42.7 (44; 38 to 49)
Mean SF-36 mental health (median; IQR)	53.6 (56.1; 51.1 to 58.8)	53.1 (55.7; 50.7 to 58.6)	53.2 (55.9; 50.6 to 58.7)	53.2 (55.6; 50.8 to 58.6)
Mean SF-36 physical health (median; IQR)	48.4 (51.3; 44.4 to 54.6)	48.1 (51.3; 44.0 to 54.8)	47.8 (50.7; 43.1 to 54.7)	48.5 (51.5; 44.8 to 54.8)
Mean CESD depression (median; IQR)	7.2 (5; 2 to 10)	7.4 (5; 2 to 10)	7.4 (5; 1 to 11)	7.4 (5; 2 to 10)
CESD > 15	16.6% (770/4646)	17.0% (328/1929)	17.6% (126/718)	17.4% (209/1199)
Mean GHQ depression & anxiety (median; IQR)	2.2 (0; 0 to 2)	2.6 (0; 0 to 2)	2.6 (0; 0 to 3)	2.4 (0; 0 to 2)
GHQ > 2/3	40.9% (1901/4646)	43.3% (835/1929)	43.0% (309/718)	43.6% (523/1199)
Mean 6 m timed walk in seconds (median; IQR)	2.4 (2.2; 1.9 to 2.7)	2.4 (2.2; 1.9 to 2.6)	2.4 (2.2; 1.9 to 2.6)	2.4 (2.2; 1.9 to 2.6)
Mean body mass index kg/m^2^ (SD)	26.6 (4.4)	27.1 (4.6)	27.2 (4.6)	27.1 (4.6)
Prescribed antihypertensives	37% (1745/4642)	35% (683/1928)	36% (258/718)	35% (417/1199)
Current smoker	7% (326/4646)	9% (166/1929)	10% (71/718)	8% (92/1199)
Mean general practitioner appointments in previous year (median; IQR)	2.9 (2; 1 to 4)	2.8 (2; 1 to 4)	2.7 (2; 1 to 4)	2.7 (2; 1 to 4)
Mean forced expiratory volume in litres (SD)	2.7 (0.8)	2.8 (0.8)	2.8 (0.7)	2.8 (0.8)
Mean mini mental state exam score (median; IQR)	28.4 (29; 28 to 29)	28.5 (29; 28 to 29)	28.5 (29; 28 to 29)	28.5 (29; 28 to 29)

Table 1 shows some demographic differences between pet owners and non-owners, but little or no difference in health variables, other than slightly worse mental health and higher BMI in owners. Pet owners were younger, more likely to still be working and married or cohabiting. They were more often in the professional/executive grade and less often in clerical/support. Pet owners reported a greater number of social activities, even after adjusting for age and retirement, and there was no difference between dogs and other pets. There was no significant association with self-reported longstanding illnesses.

### Exercise

Pet owners took significantly more mild exercise, by two MET-hours, than non-owners: median 27.8 (IQR 18.1 to 41.8) vs 25.7 (IQR 16.8 to 38.7), *p* = 0.0001. Dog owners also took significantly more, by nearly 7 MET-hours, than owners of other pets: median 32.3 (IQR 20.8 to 46.1) vs 25.6 (IQR 16.8 to 38.5), *p* < 0.0001. Moderate exercise was also significantly higher in pet owners than non-owners, by two MET-hours: median 11.8 (IQR 4.2 to 21.9) vs 9.8 (IQR 2.8 to 19.5), *p* < 0.0001. Dog owners also took significantly more moderate exercise, by 2 MET-hours, than owners of other pets: median 12.3 (IQR 4.2 to 22.2) vs 10.1 (3.1 to 20.0), *p* = 0.0002. However, there were no significant differences in vigorous exercise in MET-hours: median for pet owners 0.0 (IQR 0.0 to 4.4) vs non-owners 0.0 (IQR 0.0 to 4.4), *p* = 0.44 by Wilcoxon-Mann-Whitney test; and median for dog owners 0.8 (IQR 0.0 to 3.8) vs owners of other pets 0.0 (IQR 0.0 to 4.4), *p* = 0.48.

### The neighbourhood

We found pet owners were more positive about their neighbourhood and environment than non-owners and this was more significant with dog owners than other pets. Dog ownership significantly affects all positive outcomes associated with the area regardless of illness and age. How attached an owner feels towards their pet is linearly associated with better views of the area (Table 2).

**Table 2:** Association between pet ownership *and* perceptions of the neighbourhood: mean and median, Interquartile range (IQR)

	Non owners	Pet owners (*p*-values compared with non-owners)	Dog owners (*p*-values compared with other pets)	Cat / bird / fish / other owners	Level of attachment to the pet(s): correlation
	Mean (Median; IQR)				
I really feel part of this area	5.5 (6; 5 to 7)	5.6 (6; 5 to 7) (*p* = 0.03)	5.7 (6; 5 to 7) (*p* = 0.03)	5.5 (6; 5 to 7)	0.07 *p* = 0.0004
There is no problem with vandalism and graffiti in this area	5.3 (6; 4 to 6)	5.4 (6; 5 to 6) (*p* = 0.0002)	5.6 (6; 5 to 7) (*p* < 0.0001)	5.3 (6; 4 to 6)	0.03 *p* = 0.09
I have never felt lonely living in this area	5.6 (6; 5 to 7)	5.7 (6; 5 to 7) (*p* = 0.05)	5.8 (6; 5 to 7) (*p* = 0.01)	5.6 (6; 5 to 7)	0.02 *p* = 0.23
Most people in this area can be trusted	5.3 (6; 4 to 6)	5.5 (6; 5 to 6) (*p* = 0.002)	5.6 (6; 5 to 7) (*p* < 0.0001)	5.4 (6; 4 to 6)	0.08 *p* < 0.0001
People feel safe walking alone in this area after dark	5.2 (6; 4 to 6)	5.4 (6; 5 to 7) (*p* < 0.0001)	5.6 (6; 5 to 7) (*p* < 0.0001)	5.2 (6; 4 to 6)	0.03 *p* = 0.19
Most people in this area are friendly	5.5 (6; 5 to 6)	5.7 (6; 5 to 7) (*p* < 0.0001)	5.9 (6; 5 to 7) (*p* < 0.0001)	5.5 (6; 5 to 6)	0.10 *p* < 0.0001
People in this area will always treat you fairly	5.6 (6; 5 to 6)	5.5 (6; 5 to 6) (*p* = 0.02)	5.7 (6; 5 to 6) (*p* = 0.005)	5.5 (6; 5 to 6)	0.06 *p* = 0.002
This area is kept very clean	5.1 (5; 4 to 6)	5.2 (6; 4 to 6)	5.3 (6; 4 to 6)	5.1 (5; 4 to 6)	0.07 *p* = 0.003
		(*p* = 0.007)	(*p* = 0.009)		
If you were in trouble, there are lots of people in this area who would help you	5.0 (5; 4 to 6)	5.2 (5; 4 to 6) (*p* < 0.0001)	5.3 (5; 4 to 6) (*p* = 0.13)	5.0 (5; 4 to 6)	0.05 *p* = 0.01

### Sleep

Sleep problems were significantly correlated with poor health on three self-reported global measures (5-point Likert assessment of health in last year, 3-point assessment of health in general, binary presence of self-reported long-standing illness: *p* < 0.001 for all fifteen combinations), and there were some significant but weak correlations with less time spent in vigorous exercise.

Levels of sleep problems are shown in Table 3 for pet owners and non-owners. Pet ownership was significantly associated with less difficulty falling asleep, and with more problems waking tired.

**Table 3:** percentage of sleep problems with pet owners and non-owners. The *p*-values compare pet owners (PO) to non-pet owners (NPO)

Pet owner(PO), Non-pet owner (NPO)		Trouble falling asleep (*p* = 0.001)		Wake up several times (*p* = 0.683)		Cannot stay asleep (*p* = 0.748)		Wake as usual but feel tired (*p* = 0.010)		Disturbed sleep (*p* = 0.910)	
		PO	NPO	PO	NPO	PO	NPO	PO	NPO	PO	NPO
Frequency of problem:	Not at all	51%	46%	21%	20%	38%	37%	47%	50%	31%	30%
	1–3 days	32%	35%	27%	27%	26%	27%	26%	27%	34%	34%
	4–7 days	9%	12%	15%	16%	13%	14%	11%	11%	14%	14%
	8–14 days	4%	4%	11%	9%	8%	9%	6%	6%	8%	8%
	15–21 days	3%	2%	8%	9%	6%	6%	5%	4%	6%	6%
	22–31 days	2%	1%	18%	20%	8%	7%	5%	4%	8%	7%

Table 4 shows pet and dog effects expressed as odds ratios (ORs) for a one point worsening in reported sleep quality. We considered whether age, social activities, retirement, longstanding illness, isolation and marital status / cohabitation might have a confounding effect but found only evidence for those listed in the footnote.

**Table 4:** Association between pet ownership and sleep problems

Problem	Pet owners v Non-owners				Dog owners v owners of other pets			
	Unadjusted		Adjusted ^b^		Unadjusted^c^		Adjusted^d^	
	OR^a^ (95% CI)	*p*	OR (95% CI)	*p*	OR (95% CI)	*p*	OR (95% CI)	*p*
Trouble falling asleep	0.84 (0.76 to 0.93)	0.001	0.83 (0.75 to 0.92)	0.001	0.82 (0.69 to 0.98)	0.03	0.84 (0.69 to 1.00)	0.06
Wake several times	0.98 (0.89 to 1.08)	0.68	1.03 (0.93 to 1.13)	0.62	1.04 (0.88 to 1.23)	0.64	1.04 (0.88 to 1.24)	0.62
Cannot stay asleep	0.98 (0.89 to 1.08)	0.75	1.00 (0.91 to 1.11)	0.97	1.04 (0.88 to 1.23)	0.61	1.06 (0.89 to 1.26)	0.53
Wake as usual but feel tired	1.14 (1.03 to 1.26)	0.01	1.16 (1.04 to 1.28)	0.01	1.01 (0.85 to 1.21)	0.87	1.03 (0.86 to 1.23)	0.75
Disturbed sleep	1.01 (0.91 to 1.11)	0.91	1.01 (0.91 to 1.11)	0.88	0.98 (0.83 to 1.16)	0.79	1.00 (0.84 to 1.20)	0.96

^a^odds ratios and confidence intervals calculated from proportional odds regression models

^b^predictors in the model are binary pet ownership, age, number of social activities and longstanding illness

^c^predictors in the model are pet ownership and dog ownership

^d^predictors in the model are pet ownership, dog ownership, age, number of social activities and longstanding illness

### Mediation by exercise or perceptions of the neighbourhood

Given that this is a cross-sectional, exploratory analysis and causal relationships cannot reliably be inferred, we conducted only a basic regression to detect evidence of mediation. We compared the odds ratios seen above associating pets or dogs with either trouble falling asleep or waking tired with the same analysis adjusting for moderate exercise or feeling safe walking alone in the neighbourhood after dark.

Considering MET- hours of moderate exercise, the pets ORs changed negligibly (falling asleep: unadjusted 0.84 to 0.85, adjusted 0.83 to 0.84; waking tired: unadjusted 1.14 to 1.16,adjusted 1.16 to 1.18), and the dogs ORs likewise (falling asleep: unadjusted 0.82 to 0.82, adjusted 0.83 to 0.84; waking tired: unadjusted 1.01 to 1.02, adjusted 1.03 to 1.03).

Considering walking home after dark, the pets ORs changed negligibly (falling asleep: unadjusted 0.84 to 0.86, adjusted 0.83 to 0.85; waking tired: unadjusted 1.14 to 1.16, adjusted 1.16 to 1.16), and the dogs ORs likewise (falling asleep: unadjusted 0.82 to 0.85, adjusted 0.84 to 0.86; waking tired: unadjusted 1.01 to 1.06, adjusted 1.03 to 1.07).

### Effect modification

Among participants who are unimpaired in walking half a mile, pet ownership is associated with better views on loneliness, walking home after dark, and viewing people as friendly. Social activities are associated with all the area variables, concluding the more positive owners feel about their area the more involved they are in social activities, and self-fulfilling weekly social activities are positively affected by owning a pet although this is not significant.

We then examined whether pet-sleep effects were more pronounced for people who were less active socially and out of doors, by breaking down the sleep and pets association further with the frequency of participation in activities and hobbies (voluntary work, computers, courses and learning, cultural activities, gardening, pubs and clubs, office work, practical handiwork, religious activities, indoor games, solitary activities, visiting friends and family). First, we used Kendall’s tau-B correlation to find associations between activities and trouble falling asleep, the sleep question that shows a pet effect. There were significant but very weak correlations with computers (0.06, *p* < 0.001), cultural activities (0.03, *p* = 0.003), gardening (0.05, p < 0.001), office work (0.03, *p* = 0.010) and visiting family and friends (0.03, *p* = 0.002); in each case, people who were less active had more trouble falling asleep. It seems to be those who report never taking part in a particular activity who particularly stand out as having worse sleep, which may indicate poor underlying health. When we split the data further by pet ownership, we find no significant differences but may be losing statistical power by subdividing the data too far. Overall, we find no compelling evidence that pets are particularly beneficial for sleep in those who are more socially isolated; small correlations can be statistically significant in a large data set.

## Discussion

The contribution of this analysis to the literature is in the size of the cohort and the wide range of variables available to us. Its principal limitation is the cross-sectional nature of the comparisons because information on pet ownership was collected at one time point only.

Pet owners were on average 1.5 years younger than non-owners, as well as more likely to be married or cohabiting, less likely to be retired and involved in more social activities on average. They were more likely to be in the middle occupational grade and less likely to be in the lowest. Generic health variables did not differ much between pet owners and non-owners, and in fact there was slightly worse mental health in the pet owners. Analysis of our primary outcome showed that dog owners were able to fall asleep more easily than owners of other pets, or no pets. This may be because dog owners frequently walk their dogs just before retiring to bed and this could have a relaxing effect on the owner enabling them to fall asleep more easily. However, other aspects of sleep were not affected, except by a smaller effect participants were more likely to wake tired. This may be possibly because they were waking as a result of their pet dog, rather than waking naturally when they were refreshed from enough sleep, although adjusting for confounders weakened the association with falling asleep and removed that with waking tired. We also found an association between an active social life with friends and family and waking tired, which may explain this. Pet owners consistently felt better about their local area than non-owners, and dog owners were more positive than owners of other pets. How attached the person felt to their pets was only very weakly correlated with this.

We found no evidence that the association with the sleep outcomes was mediated either by exercise or by perceptions of the local area, and no evidence that any pet or dog ‘benefit’ was more pronounced in people who were otherwise more socially isolated.

Owning a dog requires the owner to give the dog regular exercise usually through walking around their local neighbourhood. We contend that this enables the owner to familiarise themselves with the neighbourhood, the local people at different times in the day, and enabling the pet owner to engage in exercise and sleep better after walking their dog after dark. The negative results that we saw, of the pet owner waking up tired, became borderline after adjusting for likely confounders.

There are some limitations beyond the cross-sectional analysis. Our data were collected in 2009 but we do not believe that British society has changed in any way that would undermine its external validity. Participants are required to self-complete their questionnaire two weeks before attending for the medical examination, which reduces the possibility of recall bias, and most of the questions ask for activities etc. within the last month. We have also anticipated that owning a pet or dog assumes the respondent is the carer and walker of the pet although this is not always the case [33]. Whilst we found that the majority of participants were “attached” to their pets, we have therefore assumed in our analysis the pet owner is happy to own the pet, and can afford to feed and care for their pet. We have not examined the monetary burden of owning a pet and the possible negative implications pet ownership may have. It is also necessary to note the causal relationship between findings. We found dog owners slept better but woke tired – are they waking tired because they are being woken by the dog rather than waking uninterrupted? Are our findings of worse mental health because those participants might have had worse mental health regardless of owning a dog. Similarly to Headley [15] we cannot confirm if a healthy person is more likely to get a dog, or is owning (or walking) a dog likely to make a person healthy. We found that walking a dog was associated with feeling happier about ones neighbourhood, and that non dog owners felt less safe about their neighbourhood. However, as this was a cross-sectional analysis we were limited in examining any changes over time. Headley [15] and Westgarth et al. [34] felt that pet owners needed to be distinguished from those who care for and walk the pet. In many aspects of the analysis, we have made use of batteries of questions, for example around sleep, neighbourhood and hobbies. This multiple testing increases the risk of finding an incorrectly significant result, and we regard the *p*-values as indications of the precision in the statistics and by no means as proof of a difference in the population. Whitehall II study data is representative of other white collar occupations, although not necessarily of the general population. At the point at which this data was used, this group although retired were active in part-time or voluntary work and we regard this study as focused but exploratory and hypothesis-refining. Further repeated detailed questions on sleep patterns, pet interactions and location of pet walking could inform us further.

We were limited in the number of questions we could introduce regarding pet ownership due to the already comprehensive questionnaire, and the need to avoid participant burden.

In particular, the role of sleep in the complex causal relationships among the factors has not been explored much previously, except by Headley [15] who involved a younger population. We have some limited support for Headley’s results that show dog owners sleep better, and support Reid et al. [19] by showing that older people who exercise have better quality sleep. Our results suggest that pet owners fall asleep more easily and this may be more pronounced with dog owners. We are also able to support and expand results by Cutt [14] who found dog owners felt more comfortable about their neighbourhood.

## Conclusion

In conclusion we found that pet owners found it easier to fall asleep, even after adjusting for confounders, with borderline evidence for a particular benefit from owning a dog. Pet owners consistently felt better about their local environment than non-owners, and dog owners were more positive about their environment than owners of other pets, but this did not appear to be the mechanism affecting sleep. Headley [15] recommended the use of longitudinal data to clarify directions of causal effects on this topic, and this should remain a priority.

## Abbreviations

CASP–19: Control, Autonomy, Self-realisation, Pleasure
CES – D: Centre for Epidemiologic Studies depression Scale
ELSA: English Longitudinal Study of Ageing
GHQ: General Health Questionnaire
METs: Metabolic equivalents
MMSE: Mini Mental State Examination
SF36: Short Form 36
